# Stereotactic biopsy for adult brainstem lesions: A surgical approach and its diagnostic value according to the 2016 World Health Organization Classification

**DOI:** 10.1002/cam4.4272

**Published:** 2021-09-12

**Authors:** In‐Ho Jung, Kyung Won Chang, So Hee Park, Ju Hyung Moon, Eui Hyun Kim, Hyun Ho Jung, Seok‐Gu Kang, Jong Hee Chang, Jin Woo Chang, Won Seok Chang

**Affiliations:** ^1^ Department of Neurosurgery Brain Research Institute Yonsei University College of Medicine Seoul Republic of Korea; ^2^ Department of Neurosurgery Brain Tumor Center Yonsei University College of Medicine Seoul Republic of Korea

**Keywords:** brainstem, diffuse midline glioma, medulla oblongata, midbrain, pons, Stereotactic biopsy

## Abstract

**Background:**

The brainstem has the critical role of regulating cardiac and respiratory function and it also provides motor and sensory function to the face via the cranial nerves. Despite the observation of a brainstem lesion in a radiological examination, it is difficult to obtain tissues for a pathological diagnosis because of the location and small volume of the brainstem. Thus, we aimed to share our 6‐year experience with stereotactic biopsies from brainstem lesions and confirm the value and safety of stereotactic biopsy on this highly eloquent area in this study.

**Methods:**

We retrospectively reviewed the medical records of 42 adult patients who underwent stereotactic biopsy on brainstem lesions from 2015 to 2020. The radiological findings, surgical records, pathological diagnosis, and postoperative complications of all patients were analyzed.

**Results:**

Histopathological diagnoses were made in 40 (95.2%) patients. Astrocytic tumors were diagnosed in 29 (69.0%) patients, diffuse large B cell lymphoma in 5 (11.9%) patients, demyelinating disease in 4 (9.5%) patients, germinoma in 1 (2.4%) patient, and radiation necrosis in 1 (2.4%) patient. In the 40 patients with successful stereotactic biopsy, 10 (25.0%) patients had inconsistent preoperative radiological diagnosis and postoperative pathological diagnosis. In addition, there was a difference between the treatments prescribed by the radiological and pathological diagnoses in 8 out of 10 patients whose diagnoses changed after biopsy. There was no operative mortality among the 42 patients.

**Conclusions:**

A pathological diagnosis can be made safely and efficiently in brainstem lesions using stereotactic biopsy. This pathological diagnosis will enable patients to receive appropriate treatment.

## Graphical abstract

Three approaches to stereotactic biopsy for brainstem and a Venn diagram showing the location of 42 brainstem lesion that performed stereotactic biopsy.

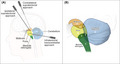

**Lay summary**

Brainstem is a very challenging area to acquire tissue for pathological diagnosis. In the current era of molecular diagnosis for brain tumors, our study demonstrated the safety and effectiveness of stereotactic biopsy to obtain tissue from adult brainstem lesions. The inconsistency rate between preoperative radiological diagnosis and postoperative pathological diagnosis reached 25%. Accurate diagnosis enables the patient to receive appropriate treatment and achieve a good prognosis.

## 1 INTRODUCTION

As the brainstem is located between the spinal cord and the brain, all neurological information passes through it. Moreover, the brainstem is the control center of vital body systems and functions such as the cardiovascular system, respiratory system, alertness, awareness, and consciousness. Because important neurological structures are concentrated in this highly eloquent region, even a small injury to the brainstem can cause severe symptoms.^1^, ^2^, ^3^, ^4^ Therefore, several surgeons hesitate to perform a biopsy even if a brainstem lesion is observed in radiological examination. In the past, the acquisition of tissue in brainstem lesions was avoided after a 1993 study reported that magnetic resonance imaging (MRI) is highly specific for diagnosing brainstem gliomas and can minimize the need for pathological confirmation.^5^

However, various pathological diagnoses can be made in this important region, from non‐neoplastic lesions such as demyelinating disease to malignant brain tumors such as glioblastoma.^6^, ^7^, ^8^, ^9^, ^10^, ^11^ In addition, it is essential to confirm the H3K27M mutation for the diagnosis of diffuse midline glioma classified as grade IV in the 2016 World health organization (WHO) classification.^12^, ^13^, ^14^, ^15^, ^16^ Molecular diagnosis, which checks for mutations that have a decisive effect on the prognosis, is impossible with radiological examination, but is possible with acquired tissues. We have previously reported that the deep brain target can be approached within 1 mm of mean positioning error through a stereotactic technique.^17^ Using this delicate technique, it is possible to safely acquire tissues from brainstem lesions. In this study, we present a large series of patients who underwent stereotactic biopsy for adult brainstem lesions. Furthermore, we discuss our 6‐year experience with stereotactic biopsies from brainstem lesions and confirm the diagnostic value and safety of stereotactic biopsy in this highly eloquent area.

## 2 METHODS

This study was conducted in accordance with the Declaration of Helsinki and was approved by the Institutional Review Board in our institute. The requirement to obtain patient's written consent was waived as this was a retrospective study.

All adult patients who underwent stereotactic biopsy of a brainstem lesion between January 2015 and December 2020 were included retrospectively. Brainstem lesions are defined as space‐occupying intra‐axial lesion involving the midbrain, pons, medulla oblongata, or cerebellar peduncle. Stereotactic biopsy for brainstem lesions performed before 2015 were excluded because tests for the H3K27M mutation to diagnose diffuse midline gliomas were not performed.

### 2.1 Surgical technique and approach

All patients were mounted on the Leksell stereotactic frame G (Elekta Instruments AB) under local anesthesia on the day of the surgery. After the stereotactic frame was fixed to the head, the patients underwent MRI (1.5 Tesla Philips Achieva). MRI sequences for stereotactic biopsy included gadolinium‐enhanced T1‐weighted images with a slice thickness of 1.5 mm and T2‐weighted images with a slice thickness of 2.5 mm. Of the ipsilateral supratentorial, contralateral supratentorial, or infratentorial transcerebellar approaches, we preferred the supratentorial approach for cephalic brainstem (e.g., midbrain) lesions. In the caudal brainstem lesions (e.g., medulla oblongata, cerebellar peduncle), the infratentorial transcerebellar approach was used when the trajectory of the supratentorial approach was disturbed by the tentorium. However, in addition to considering the target location and tentorium in determining the approach, neuroanatomy, vessels, ventricle, and the patient neck were also considered. To prevent neurologic deficit after biopsy, we tried not to damage important neuroanatomical structures such as the oculomotor nucleus, medial longitudinal fasciculus, red nucleus, abducens nucleus, and facial nucleus. In addition, the trajectory was designed to avoid the ventricle and vessels that could be observed in MRI. The infratentorial transcerebellar approach uses a low entry point to avoid tentorium. Therefore, for the trajectory not to be disturbed by the posterior bar of the Leksell frame, the frame should be fixed as low as possible. In addition, the transcerebellar approach was performed in the prone or semi‐sitting position. Since both positions require considerable neck flexion, the flexibility of the patient's neck should also be considered. The target point, entry points, and trajectory of each biopsy were designed using SurgiPlan (Elekta instruments AB).

The procedure was performed under general anesthesia with the Sedan side‐cutting biopsy needle, which has a 5‐mm window (Elekta instruments AB). We tried to collect tissue from the four directions at as many points as possible within the same trajectory to secure enough tissue to improve the diagnosis rate. However, if the tumor was too small to set multiple harvest points, samples were collected only at the target point. Postoperative computed tomography (CT) scans were taken in all patients to assess potential hemorrhage at the biopsy site.

### 2.2 Outcome of stereotactic biopsy for brainstem lesion

The histopathological diagnosis success rate, mortality, and morbidity were investigated after stereotactic biopsy for brainstem lesions. In addition, patients with discrepancies between preoperative radiological diagnosis and postoperative pathological diagnosis were examined in detail.

### 2.3 Statistical analysis

For statistical analysis, the IBM SPSS Statistics (Version 25.0; IBM) was used. The Student's *t*‐test was used for statistical comparisons between groups. *p*‐values <0.05 were considered statistically significant.

## 3 RESULTS

### 3.1 Patient demographics and tumor characteristics

**FIGURE 1:**
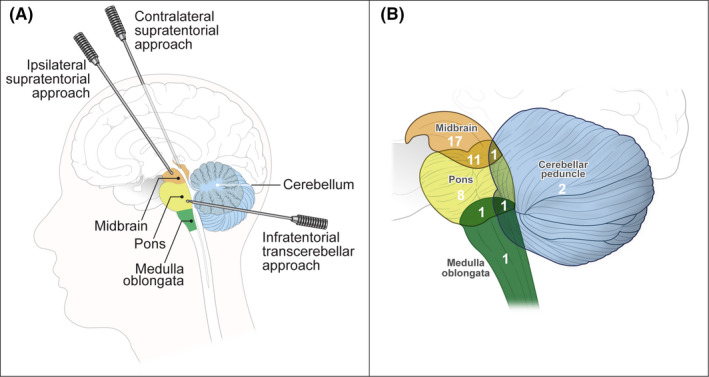
(A) Three approaches to stereotactic biopsy for brainstem lesions. (B) A Venn diagram showing the location of 42 brainstem lesions subjected to stereotactic biopsy

Forty‐two adult patients underwent stereotactic biopsy for their brainstem lesions from January 2015 to December 2020 in our institution. Twenty‐four patients were female and 18 were male. The mean age was 49.4 years. The average maximal diameter of the tumor was 2.2 cm. The lesions involved the midbrain in 29 (69.0%) patients, the pons in 22 (52.4%) patients, the medulla oblongata in 3 (7.1%) patients, and the cerebellar peduncle in 4 (9.5%) patients (Figure 1). Fourteen (33.3%) patients needed surgical treatment due to hydrocephalus. The demographics of the patients and the tumor characteristics are detailed in Table 1.

**TABLE 1:** Demographics and tumor characteristics

Category	*n* = 42
Sex	
Female	24 (57.1%)
Male	18 (42.9%)
Age	49.4 ± 16.0
Karnofsky performance score	
70	11 (26.2%)
80	11 (26.2%)
90	18 (42.9%)
100	2 (4.8%)
Brainstem involvement	
Midbrain	29 (69.0%)
Pons	22 (52.4%)
Medulla oblongata	3 (7.1%)
Cerebellar peduncle	4 (9.5%)
Hydrocephalus	14 (33.3%)
Evans’ index	0.279 ± 0.044
Maximal diameter (cm)	2.2 ± 0.9
Location	
Left	21 (50.0%)
Right	13 (31.0%)
Central	8 (19.0%)

The most common symptom was diplopia (12 patients, 28.6%), followed by hemiparesis (10 patients, 23.8%) and hemiparesthesia (7 patients, 16.7%) (Table 2).

**TABLE 2:** Symptoms of patients who underwent stereotactic biopsy for brainstem lesion

Category	*n* = 42
Diplopia	12 (28.6%)
Hemiparesis	10 (23.8%)
Hemiparesthesia	7 (16.7%)
Dizziness	5 (11.9%)
Headache	5 (11.9%)
Dysarthria	4 (9.5%)
Gait disturbance	3 (7.1%)
Facial numbness	2 (4.8%)
Impaired cognitive function	1 (2.4%)
Quadriparesis	1 (2.4%)
Loss of taste	1 (2.4%)
Tremor	1 (2.4%)
Dyskinesia	1 (2.4%)
Asymptomatic	2 (4.8%)

### 3.2 Stereotactic biopsy

Stereotactic biopsy was performed with the ipsilateral supratentorial approach in 34 (81.0%) patients, contralateral supratentorial approach in 6 (14.3%) patients, and infratentorial transcerebellar approach in 2 (4.8%) patients. The average Evans’ index of the patients was 0.279 (standard deviation, 0.044; range, 0.193–0.394). The average distance from the midline to the target point was 8.6 mm, and the average distance from midline to entry point was 42.3 mm. Ten (23.8%) patients already had hydrocephalus that needed surgical treatment before biopsy. Fourteen (33.3%) patients underwent surgical treatment for hydrocephalus (Table 3).

**TABLE 3:** Outcome of stereotactic biopsy for brainstem lesions

Category	
Trajectory	
Ipsilateral supratentorial	34 (81.0%)
Contralateral supratentorial	6 (14.3%)
Infratentorial transcerebellar	2 (4.8%)
Target laterality (mm)	8.6 ± 5.8
Entry laterality (mm)	42.3 ± 13.0
Number of acquired tissues	4.7 ± 2.1
Pathologic diagnosis rate	40/42 (95.2%)
Radio‐Pathologic discordance rate	10/40 (25.0%)
Mortality	0 (0.0%)
Morbidity	
Transient	3 (7.1%)
Permanent	1 (2.4%)

The radiological characteristics between the patients who had stereotactic biopsy performed using the ipsilateral supratentorial approach and those using the contralateral supratentorial approach were compared. There were no significant differences in the Evans’ index, target laterality, and entry laterality between the two approaches (Table 4).

**TABLE 4:** Comparison of ipsilateral supratentorial approach and contralateral supratentorial approach

Radiological characteristics	Approach		*p*
	Ipsilateral supratentorial (*N* = 34)	Contralateral supratentorial (*N* = 6)	
Evans’ index	0.279 ± 0.040	0.292 ± 0.062	0.507
Target laterality (mm)	7.9 ± 5.9	9.9 ± 4.6	0.429
Entry laterality (mm)	42.3 ± 10.3	48.7 ± 20.8	0.497

Operative mortality did not occur in our series. Transient morbidity occurred in three (7.1%) patients. Mild dysarthria and hemiparesis occurred in two patients with focal hemorrhage at the biopsy site. However, symptoms improved within 1 month. Postoperative epidural hemorrhage requiring surgical treatment occurred in one patient. The patient recovered completely after surgical treatment. Permanent morbidity occurred in one (2.4%) patient. Hemorrhage at the biopsy site was observed on the patient's postoperative CT. The patient showed decreased consciousness and severe hemiplegia. After 2 months of intensive care, the patient was transferred to another hospital without full recovery (Table 3).

### 3.3 Histopathological diagnosis

**FIGURE 2:**
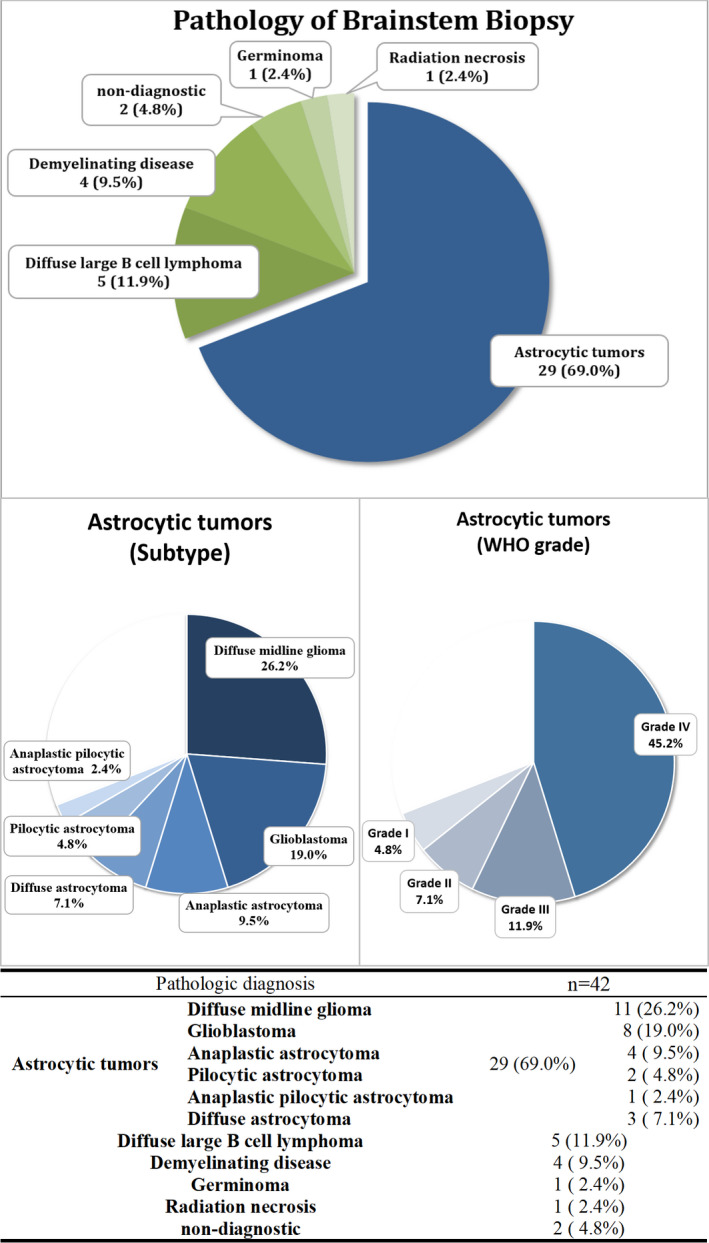
Histopathological diagnoses of brainstem lesions. Astrocytic tumors were diagnosed in 29 (69.0%) patients, diffuse large B cell lymphoma in 5 (11.9%) patients, demyelinating disease in 4 (9.5%) patients, germinoma in 1 (2.4%) patient, and radiation necrosis in 1 (2.4%) patient. Among the astrocytic tumors, diffuse midline glioma (11 patients, 26.2%) was the most common, and glioblastoma (8 patients, 19.0%) was the second most common. Among the astrocytic tumors located in the brainstem, 19 (45.2%) were grade IV, 5 (11.9%) were WHO grade III, 3 (7.1%) were WHO grade II, and 2 (4.8%) were WHO grade I as per the 2016 WHO Classification. WHO, World Health Organization

The histopathological diagnosis success rate of stereotactic biopsy for brainstem lesions was 95.2% (40 out of 42 patients). Two (4.8%) patients did not receive pathological diagnoses due to the inadequate tissue sampling. Astrocytic tumors were diagnosed in 29 (69.0%) patients, diffuse large B cell lymphoma in 5 (11.9%) patients, demyelinating disease in 4 (9.5%) patients, germinoma in 1 (2.4%) patient, and radiation necrosis in 1 (2.4%) patient. Among the patients with astrocytic tumors, 11 (19.0%) exhibited the H3K27 mutation; thus, diffuse midline glioma was the most common lesion, followed by glioblastoma (8 patients, 19.0%). Among the astrocytic tumors located in the brainstem, 19 (45.2%) were grade IV, 5 (11.9%) were grade III, 3 (7.1%) were grade II, and 2 (4.8%) were grade I as per the 2016 WHO Classification. Histopathological diagnoses of the brainstem lesions are detailed in Figure 2.

**FIGURE 3:**
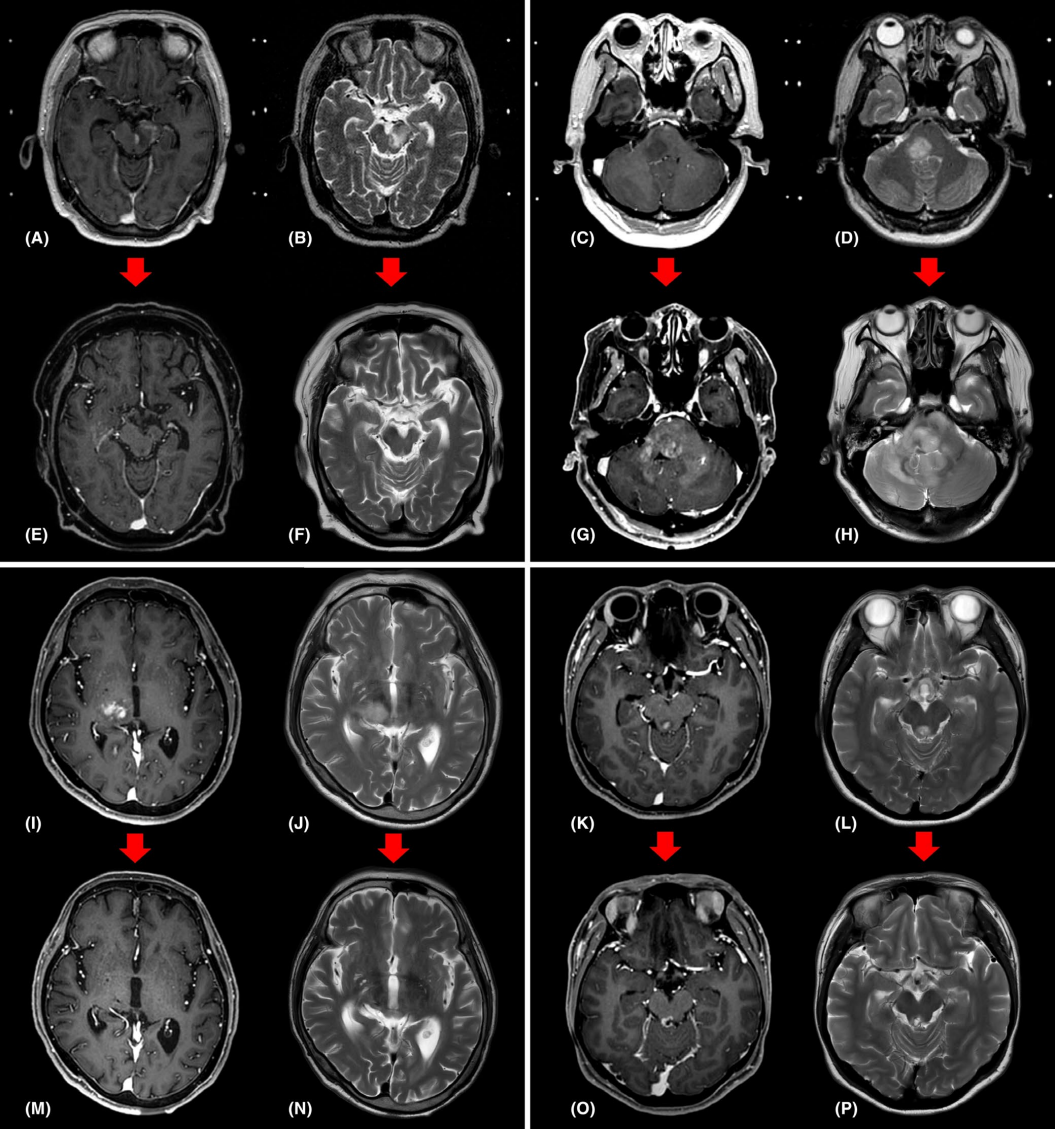
MRI findings for two cases with different radiological and pathological diagnoses and two cases with same radiological and pathological diagnoses. (A, B) T1‐weighted image with gadolinium enhancement and T2‐weighted image of a 62‐year‐old woman complaining of hemiparesis. A T2 hyperintense lesion with subtle focal enhancement is located in the left midbrain. Radiologically, low grade glioma with high‐grade component was suspected, but it was diagnosed as a demyelinating disease through stereotactic biopsy. (E, F) The patient was treated with steroids, and the MRI taken 3 months later showed improvement of the lesion. (C, D) T1‐weighted image with gadolinium enhancement and T2‐weighted image of a 50‐year‐old woman complaining of dizziness. An infiltrative T2 hyperintense lesion without enhancement is located in the right pons and medulla. Radiologically, low grade glioma was suspected, but diffuse midline glioma was diagnosed through stereotactic biopsy. (G, H) Despite concurrent chemoradiation therapy, the MRI taken 2 years later showed disease progression. (I, J) T1‐weighted image with gadolinium enhancement and T2‐weighted image of a 72‐year‐old woman complaining of hemiparesis. A T2 high signal intensity lesion with enhancement is located in the right thalamus, midbrain, and pons. Although lymphoma was suspected by radiology, it was necessary to exclude demyelinating lesion or high‐grade glioma. Diffuse large B cell lymphoma was confirmed through stereotactic biopsy, and chemotherapy (Methotrexate, Vincristine, and Dexamethasone) was initiated. (M, N) The enhancing lesion disappeared on follow‐up MRI after 6 months. (K, L) T1‐weighted image with gadolinium enhancement and T2‐weighted image of a 43‐year‐old woman complaining of diplopia. A contrast‐enhancing lesion is observed in the midbrain. High‐grade glioma was suspected by radiology, and pathological diagnosis was attempted through stereotactic biopsy to determine the treatment. After confirmation of anaplastic astrocytoma, the patient underwent radiation therapy. (O, P) And there was no significant change in follow‐up MRI after 6 months. MRI, magnetic resonance imaging

Of the 40 patients for whom a pathological diagnosis was made, 10 (25.0%) patients showed inconsistency between the preoperative radiological diagnosis and the postoperative pathological diagnosis (Tables 3, and 5). The grade of glioma was changed in six cases (Figure 3C,D,G,H). In two cases, the diagnosis was changed to other tumor types rather than the expected one. In the other two cases, the diagnosis was changed to demyelinating disease rather than malignancy (Figure 3A,B,E,F). In addition, there was a difference between the treatments prescribed by the radiological and pathological diagnoses in 8 out of 10 patients whose diagnoses changed after biopsy (except for patient number 2 and patient number 24, Table 5). In other words, out of the 40 patients whose pathological diagnoses were made by stereotactic biopsy, eight (20%) had a change in their treatment plan.

**TABLE 5:** Patients inconsistent between preoperative radiological diagnosis and postoperative pathologic diagnosis

No	Op date	Sex	Age	Location	Size (cm)	Rt/Lt	Trajectory	Radiologic diagnosis	Pathologic diagnosis	Treatment
1	2015‐03‐19	F	62	Midbrain	1.5	Lt	Ipsilateral supratentorial	Low grade glioma	Demyelinating disease	Steroid pulse therapy
2	2015‐05‐22	F	47	Pons, medulla, Cbll peduncle	2.9	Lt	Contralateral supratentorial	Low grade glioma	Anaplastic astrocytoma	RTx
8	2016‐09‐07	F	49	Cbll peduncle	1.3	Rt	Intratentorial transcerebellar	Low grade glioma with focal high grade	Demyelinating disease	Steroid pulse therapy
11	2017‐02‐03	M	41	Midbrain	1.8	Lt	Ipsilateral supratentorial	High grade glioma	Germinoma	RTx
12	2017‐05‐22	M	42	Midbrain, pons	2.5	Rt	Ipsilateral supratentorial	Low grade glioma	Diffuse midline glioma	CCRT
14	2017‐06‐16	M	61	Pons	2.2	Rt	Ipsilateral supratentorial	Metastasis	Glioblastoma	CCRT
20	2018‐05‐29	F	50	Pons, medulla	2.7	Rt	Contralateral supratentorial	Low grade glioma	Diffuse midline glioma	CCRT
24	2018‐12‐18	F	60	Midbrain, pons	4.4	Lt	Ipsilateral supratentorial	Low grade glioma	Anaplastic astrocytoma	Follow‐up loss
30	2019‐10‐07	F	48	Midbrain, pons	3.5	Rt	Ipsilateral supratentorial	Low grade glioma	Diffuse midline glioma	CCRT
38	2020‐07‐23	F	30	Midbrain, pons	3.2	Lt	Ipsilateral supratentorial	High grade glioma	Pilocytic astrocytoma	RTx

Abbreviations: Cbll, cerebellar; CCRT, concurrent chemoradiotherapy; F, female; Lt, left; M, male; Op, operation; Rt, right; RTx, radiation therapy.

## 4 DISCUSSION

There are several diseases that can occur in the brainstem. They range from high‐grade glioma with a catastrophic prognosis despite aggressive treatment, to non‐neoplastic disease that does not require chemotherapy or radiation therapy. Therefore, it is important to make an accurate diagnosis for brainstem lesions, as treatments that can affect the prognosis change drastically depending on the diagnosis. Acquiring the tissue of the lesion is most accurate to make a pathological diagnosis. However, because of the location of the brainstem and there are important neurological structures are concentrated in its small volume, it is difficult to obtain tissues through biopsy. Therefore, research has been conducted to understand whether radiological diagnosis can replace pathological diagnosis, and the necessity of biopsy for brainstem lesions has been controversial.

### 4.1 Discordance between radiological diagnosis and pathological diagnosis

Some studies have reported that brain MRI shows high sensitivity and specificity when diagnosing brainstem lesions without biopsy. Therefore, they argued that the need for histological confirmation could be omitted when MRI, clinical history, and laboratory data are considered together. There are even claims that tissue diagnosis did not alter the therapy in diffuse brainstem glioma.^5^, ^18^, ^19^ Additional information provided by magnetic resonance spectroscopy, another non‐invasive tool, can also help in discriminating brainstem lesions.^20^, ^21^, ^22^, ^23^

However, other studies argue that diagnosis of brainstem lesions made by radiological examination alone is unreliable. Rachinger et al. reported that the specificity of MRI was 46.6% and sensitivity was 62.5% in patients with low grade gliomas (WHO grades I and II), and the specificity was 61.7% and sensitivity was 58.3% in patients with high‐grade gliomas (WHO grades III and IV).^24^ Hankinson et al. invited pediatric neurosurgeons to conduct a survey study using MR images from 16 children with diffuse pontine tumors. The median percentage of respondents who disagreed with the majority opinion regarding whether a tumor qualified as typical was 28.6%. More than 75% agreement regarding whether a tumor was typical or atypical was found in only 43.8%.^25^

In our study, out of the 40 patients with brainstem lesions for whom pathological diagnosis was made through stereotactic biopsy, 10 patients showed differences in preoperative radiological diagnosis and postoperative pathological diagnosis. In addition, since there was a difference between the treatment prescribed by the radiological and pathological diagnoses in eight patients, tissue confirmation through stereotactic biopsy provided valuable information for these patients. As mentioned earlier, in the studies conducted in the 1990s and 2000s, radiological diagnoses of brainstem lesions through brain MRI were able to predict the pathological diagnosis adequately.^5^, ^18^, ^19^ However, recent studies, including our study, showed differences between the radiological diagnosis and the pathological diagnosis. The accuracy of radiological diagnosis has decreased recently compared to the past because of the emergence of diffuse midline glioma. Diffuse midline glioma has been defined as a midline located glioma with predominately astrocytic differentiation, harboring a K27M mutation in either H3F3A or HIST1H3B/C. The presence of the K27M mutation in midline‐located glioma are classified as WHO grade IV even in the absence of other morphologic high‐grade features.^12^, ^13^, ^14^, ^15^, ^16^, ^26^, ^27^ Therefore, although MRI and histologic findings suggest a low‐grade glioma feature, there are cases where the final diagnosis using molecular data are diffuse midline gliomas, which are high‐grade gliomas. In our study, three patients were predicted to be low grade glioma by radiological diagnoses, but were finally diagnosed with diffuse midline glioma by tissue confirmation.

### 4.2 Stereotactic biopsy technique

The tissue obtained through a stereotactic biopsy is a very small part of the entire lesion. Therefore, in heterogeneous tumors, there is a concern that tissue obtained through biopsy may not represent the pathology of the entire tumor.^28^, ^29^ To overcome the shortcomings of stereotactic biopsy, we tried to collect tissue in four directions at as many points as possible within the same trajectory. Accordingly, we were able to reflect heterogeneity of the tumor and increase the success rate of stereotactic biopsy diagnosis. The diagnostic success rate of our study was 95.2%. This is comparable to the average diagnostic success rate of pediatric brainstem tumor of 96.6% reported by Hamisch et al. through a meta‐analysis of 735 cases.^8^

Of the three approaches to reach the brainstem lesion in stereotactic biopsy, we preferred the supratentorial approach for cephalic brainstem lesions. However, in patients with brainstem lesions, the supratentorial trajectory may be disturbed by enlarged lateral ventricle due to hydrocephalus. In our study, the average Evans’ ratio of patients was 0.279, which is greater than the normal value.^30^ The trajectory should not ideally penetrate the ventricle. Trajectory penetrating the ventricle has two drawbacks. First, the number of pial and ependymal surfaces that such a trajectory passes through increases, which can damage the choroid plexus or subependymal vessels and cause intracranial hemorrhage. Second, brain shifting may occur due to cerebrospinal fluid loss. Because the shifting changes the location of the target, it may cause failure to obtain the target tissue in stereotactic biopsy.^31^, ^32^, ^33^ To avoid ventricle penetration, we moved the entry point of the supratentorial approach laterally, or used a contralateral approach instead of ipsilateral. Therefore, the average Evans’ index of the contralateral supratentorial approach was higher than that of the ipsilateral supratentorial approach in our study. The average entry point laterality of the contralateral supratentorial approach was higher than that of the ipsilateral supratentorial approach. However, neither of them showed statistical difference due to the small sample size.

In lesions of the caudal brainstem, the tentorium may interfere with the trajectory of supratentorial approach. In this case, the contralateral supratentorial approach or the infratentorial transcerebellar approach can be used. In the infratentorial transcerebellar approach, the higher the location of the lesion and the steeper the tentorium slope, the lower is the entry point needed to prevent the trajectory from being disturbed by the tentorium. Therefore, to avoid disturbance of the trajectory by the posterior bar of the Leksell frame, the frame should be fixed as low as possible. However, if the patient has a short neck and high shoulders, the infratentorial transcerebellar approach cannot be used as the Leksell frame interferes with the transcerebellar trajectory. As mentioned in the Method section, there is no absolute criterion for selecting an approach to the brainstem lesion. The most appropriate approach should be determined for each patient, considering the locations of the lesion and the tentorium, the neuroanatomy, vessels, ventricle, and idiosyncrasies of the patient neck.

It has been previously reported that brainstem lesion biopsy has high morbidity and high mortality. This risk has led many neurosurgeons to hesitate to acquire tissue from brainstem lesions.^34^, ^35^, ^36^ However, it has been recently reported that biopsies from brainstem lesions are as safe as biopsies from other regions of the brain.^6^, ^7^, ^8^, ^37^ In a single institution study, Puget et al. reported a 100% diagnostic yield in 130 pediatric patients of diffuse intrinsic pontine glioma who underwent stereotactic biopsy. They did not observe any mortality or permanent deficit; however, a transient deterioration of neurological deficit occurred in five patients (3.9%).^37^ Hamisch et al. reported a 96.1% diagnostic success rate, 0.6% mortality, 6.7% overall morbidity, and 0.6% permanent morbidity in a meta‐analysis of 735 cases of pediatric brainstem tumors.^8^

Recently, with the advancement of neuronavigation technologies, frameless stereotactic brain biopsy and robot‐assisted stereotactic brain biopsy have been introduced.^38^, ^39^, ^40^, ^41^ Recent studies have reported that the stability and efficiency of these new technologies are equivalent to those of standard frame‐based stereotactic biopsy. We have also started using robot‐assisted surgery to implant electrodes in patients with epilepsy, with accuracy and safety similar to those of conventional stereotactic procedures. These new technologies are expected to facilitate easy and safe brainstem biopsy, and we look forward to applying these new technologies to perform biopsy from such critical structures.

In the era of molecular diagnosis for brain tumors, our study demonstrated the safety and effectiveness of stereotactic biopsy to obtain tissue from adult brainstem lesions. In 42 adult patients with brainstem lesion, the diagnostic success, mortality, and permanent morbidity rates were 95.2%, 0.0%, and 2.4%, respectively. Therefore, adult brainstem lesions can be safely and efficiently diagnosed through stereotactic biopsy similar to pediatric brainstem lesions.

### 4.3 Limitations

Our study is limited by its retrospective study design and small sample size. Moreover, because of this, we failed to show statistical differences between approaches. If more cases are included in further studies, more meaningful results can be obtained. In addition, although this study was conducted on all consecutive patients with brainstem lesions who underwent stereotactic biopsy, stereotactic biopsy was not performed on all patients with brainstem lesions who visited the institution. Therefore, our pathological diagnosis may not accurately represent a brainstem lesion.

## 5 CONCLUSIONS

In summary, this study presents a large series of patients who underwent stereotactic biopsy for adult brainstem lesions. The findings suggested that safe and effective diagnosis of adult brainstem lesions is possible using stereotactic biopsy. In addition, 25.0% of the patients showed an inconsistency between the preoperative radiological diagnosis and the postoperative pathological diagnosis, and 20.0% changed their treatment plan because of their pathological diagnosis. Therefore, through successful stereotactic biopsy, appropriate treatment may be provided to patients with brainstem lesions by selecting a proper approach.

## CONFLICT OF INTEREST

The authors have nothing to disclose.

## ETHICAL APPROVAL

This study was conducted in accordance with the Declaration of Helsinki and was approved by the Institutional Review Board in our institute. The requirement to obtain patient's written consent was waived as this was a retrospective study.

## Data Availability

The data that support the findings of this study are available from the corresponding author upon reasonable request.
